# Imported malaria in adults: about a case of cerebral malaria

**DOI:** 10.1186/s41935-022-00279-1

**Published:** 2022-04-01

**Authors:** Sarra Ben Abderrahim, Sarra Gharsallaoui, Amal Ben Daly, Amal Mosbahi, Selma Chaieb, Zeineb Nfikha, Samar Ismaïl, Chahnez Makni, Moncef Mokni, Akila Fathallah-Mili, Maher Jedidi, Mohamed Ben Dhiab

**Affiliations:** 1 Ibn El Jazzar Faculty of Medicine, the University of Sousse, Mohamed Karoui street, 4002 Sousse, Tunisia; 2 Department of Forensic Medicine, Farhat Hached University Hospital, Ibn El Jazzar street, 4000 Sousse, Tunisia; 3 Department of Pathological Anatomy and Cytology, Farhat Hached University Hospital, Ibn El Jazzar street, 4000 Sousse, Tunisia; 4 Parasitology-Mycology Laboratory, Farhat Hached University Hospital, Ibn El Jazzar street, 4000 Sousse, Tunisia

**Keywords:** Malaria infection, Cerebral malaria, Forensic medicine, Autopsy, Postmortem, Tunisia

## Abstract

**Background:**

Malaria is the first parasitic infection endemic in the world caused by parasites species of *Plasmodium*. Cerebral malaria (CM) is a rapidly progressive and severe form of *Plasmodium falciparum* infection, characterized by a greater accumulation of red blood cells parasitized by *Plasmodium falciparum* in the brain. The diagnosis of malaria is usually made in living patients from a blood sample taken in the course of a fever on return from an endemic country, whereas CM, often associated with fatal outcomes even in treated subjects, is usually diagnosed at autopsy.

**Case presentation:**

We present the case of a 36-year-old man who died a few days after returning from a business trip to the Ivory Coast. As a result of an unclear cause of death, a medicolegal autopsy was ordered. Autopsy findings revealed massive congestion and edema of the brain with no other macroscopic abnormalities at organ gross examination. Histology and laboratory tests were conducted revealing a *Plasmodium falciparum* infection, with numerous parasitized erythrocytes containing dots of hemozoin pigment (malaria pigment) in all examined brain sections and all other organs. Death was attributed to cerebral malaria with multiple organ failure.

**Conclusions:**

This report summarizes several features for the diagnosis of malaria and how postmortem investigations, as well as histology and laboratory diagnosis, may lead to a retrospective diagnosis of a fatal complicated form with cerebral involvement.

## Background

Imported malaria in adults is an infection caused by parasites species of *Plasmodium* (single-celled parasites) that occurs in a person returning from a stay in a malaria-endemic area (Bruneel et al. 2012). Since its eradication in 1979 (Chahed et al. 2001), Tunisia has stopped the chain of indigenous malaria transmission. All cases reported in Tunisia since that date are imported cases (Ayadi et al. 2000; Belhadj et al. 2008; Aoun et al. 2010; Mtibaa et al. 2018). This is mainly due to the increasing number of African students who come to Tunisia for their studies and the growing international commercial and professional exchanges with malaria-affected African countries (Belhadj et al. 2008). The vast majority of severe malaria attacks are related to *Plasmodium falciparum* (among the four pathogenic agents of human malaria), due to its neurological damage, known as “cerebral malaria (CM)” or “neuromalaria” (Pongponratn et al. 2003). In adults, CM is often part of a picture of multi-visceral failure. This condition is associated with a large number of sequestered parasites throughout the organs of the body (especially the brain), and disseminated intravascular coagulation, rapidly leading to death (Milner et al. 2015). Convulsions are though less frequent than in children (Milner et al. 2015). The diagnosis of malaria is usually made in living patients from a blood sample taken in the course of a fever on return from an endemic country. Our observation constitutes one of the rare cases where the diagnosis was made only postmortem, emphasizing the need for cross-disciplinary collaboration in the diagnosis of CM after death.

## Case presentation

**Fig. 1:**
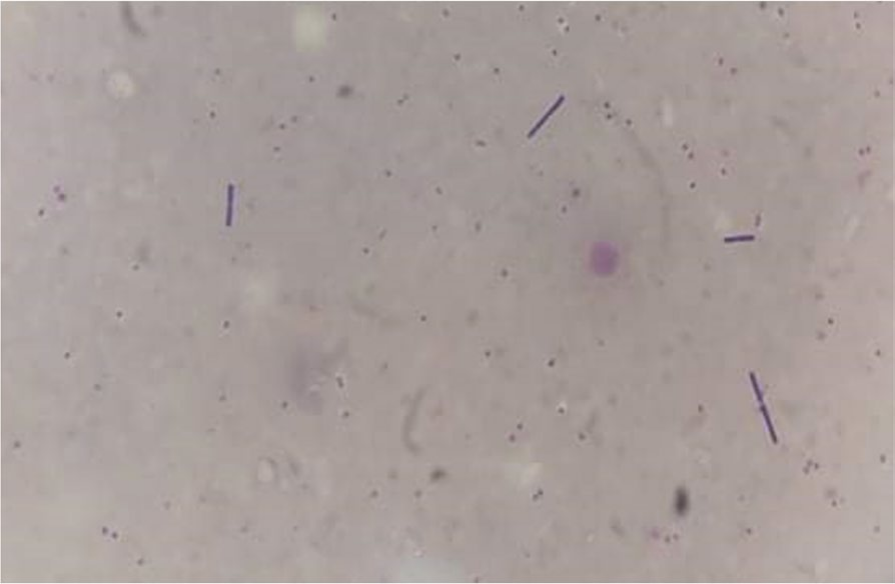
Thick blood smear showing numerous *Plasmodium falciparum*. Giemsa, ×100

**Fig. 2:**
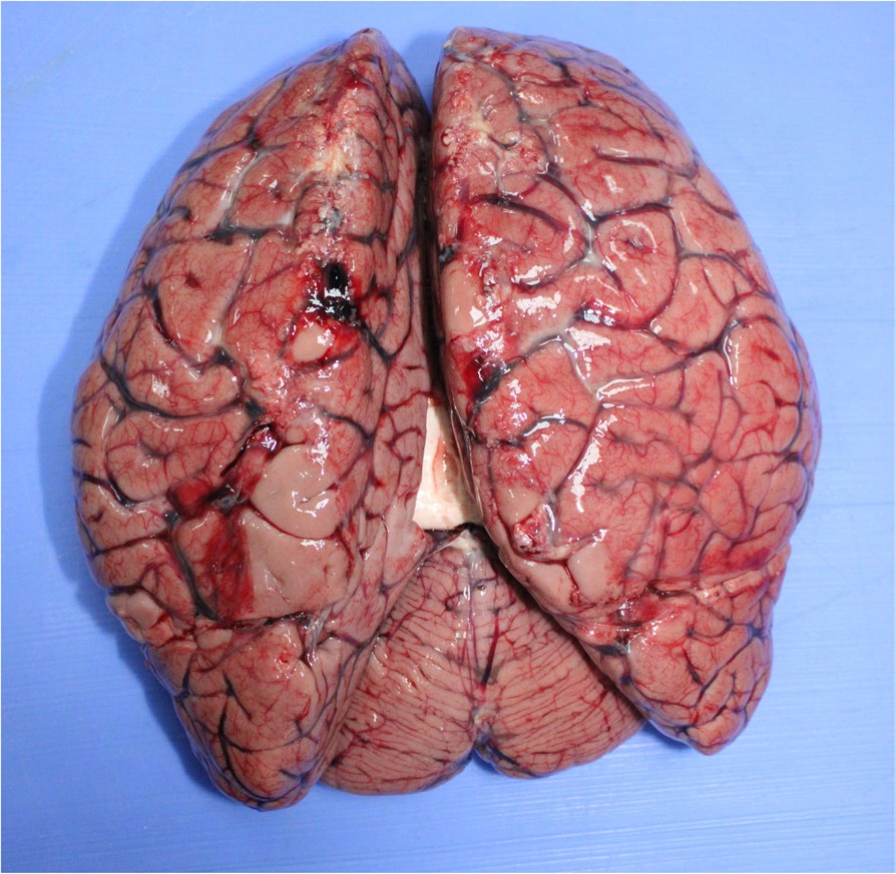
Representative image of the brain at autopsy showing congestion and edema

**Fig. 3:**
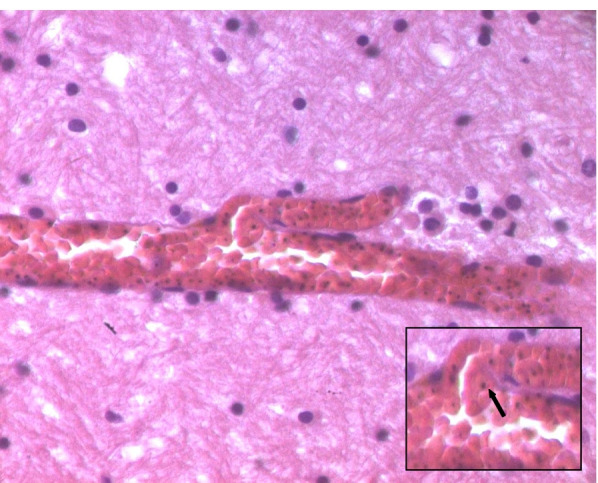
Histology findings showing congested brain capillary containing numerous parasitized erythrocytes (trophozoites), with intraerythrocytic malaria pigment appearing as small black dots (enlarged image with arrow). Hematoxylin and eosin, ×400

**Fig. 4:**
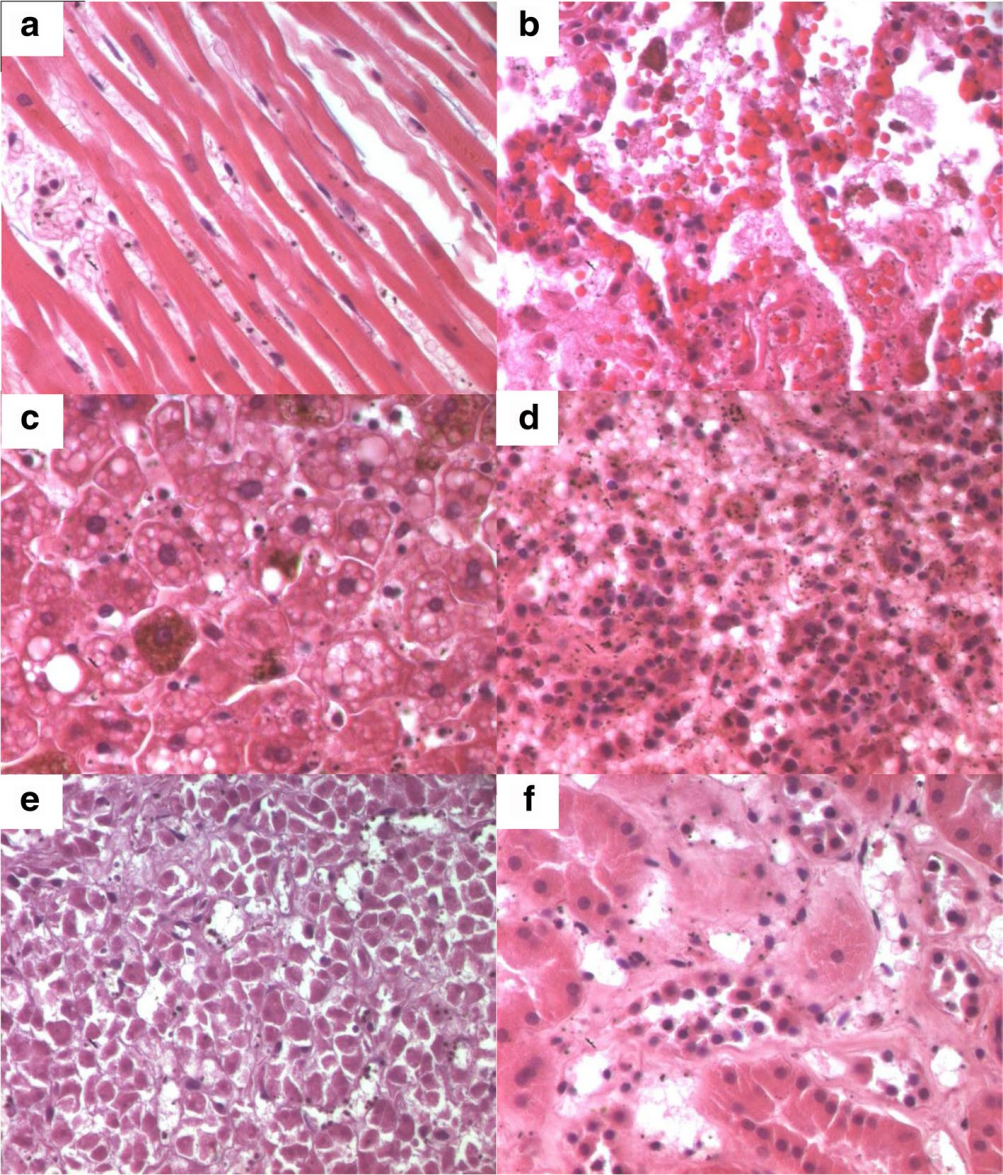
Histology findings showing numerous parasitized erythrocytes (trophozoites) of the organs, 400× original magnification. **a**–**f** Hematoxylin and eosin-stained sections of the heart (**a**), lung (**b**), liver (**c**), spleen (**d**), pancreas (**e**), and kidney (**f**)

The case involves Mr. N., a 36-year-old man with no previous medical history, who had returned from a long stay in Ivory Coast for professional activities (about 0–6 months). Since his return to Tunisia, he presented a symptomatology associating fever, headache, asthenia, and diarrhea. A *polymerase chain reaction* (PCR) test for Covid-19 had previously been performed (before traveling), which was negative. Mr. N. did not seek medical advice and resorted to self-medication including antipyretic and analgesics. On the 5th day, Mr. N.’s condition worsened as he presented a generalized tonic-clonic convulsive seizure, after which he did not regain consciousness. His family members called for medical assistance, but Mr. N. died before the arrival of the emergency units. As a result of an unclear cause of death, a medicolegal autopsy was ordered by the prosecutor, and the body was thus transferred to our department for autopsy. Malaria infection was already suspected at this point upon investigating the death circumstances with his relatives. A postmortem blood sample, on ethylenediamine tetraacetic acid (EDTA) preserved blood sample, was hence referred to the Parasitology Laboratory in order to confirm our assumptions before autopsy. Malaria rapid diagnostic test (RDT) (i.e., rapid diagnostic test for malaria which detects malaria antigens in a person’s blood) was positive. In both thick and thin Giemsa-stained blood smears (Fig. 1), numerous *Plasmodium falciparum* (trophozoites) were found, showing high parasitemia. The percentage of parasitemia was though difficult to assess due to the hemolysis state of the postmortem samples. External examination revealed cyanosis with frank mucocutaneous icterus. Autopsy findings revealed massive congestion and edema of the brain, weighing 1550 g (Fig. 2), lungs (weighing 745 g on the right and 640 g on the left), and inner organs. Hepatomegaly was noted (liver weighing 2905 g) with heterogeneous parenchyma on section. The spleen (weighing 385 g) was enlarged with a tense, smooth capsule, and congested parenchyma of brown-black color. Histology showed cerebral gray and white matter with congested capillaries (Fig. 3) containing numerous parasitized erythrocytes (trophozoites) in each examined brain section. Each cell contained dots of hemozoin pigment (malaria pigment). The same findings were also observed in the histology of the remaining thoracic and abdominal organs (Fig. 4). Toxicological analyses did not reveal the presence of any toxic substance that might have been involved in the death.

Based on histology, autopsy findings, as well as parasitology expertise, the case was considered to have died of fulminant cerebral malaria with multiple organ failure.

## Discussion

In the present case, a postmortem diagnosis of cerebral malaria with multiple organ failure due to malaria infection was observed. Similar autopsy diagnoses of undiagnosed or misdiagnosed malaria cases with cerebral involvement were described in the literature (Kain et al. 2001; Muehlethaler et al. 2005; Alunni-Perret et al. 2010; Checkley et al. 2012; Ajili et al. 2013; Yağmur et al. 2015; Féron et al. 2017)*.* To the best of our knowledge, the reported case is one of the rarest autopsy discoveries of CM in Tunisia. As the term implies, imported neuromalaria corresponds to imported cases of acquired exposure to *Plasmodium* parasites in endemic regions, to non-endemic countries (Mischlinger et al. 2020). There are many reasons which can lead to this importation. The main sources include traditional cross-border migration in search of work, tourists coming back from endemic countries, displacement due to demographic changes (Odolini et al. 2012; Sriwichai et al. 2017), and even in military personnel returning from an external operation (Ajili et al. 2013). Our case corresponded to imported malaria during travel undertaken for professional reasons.

Cerebral malaria, renal malaria, and pulmonary malaria are the three most common causes of sudden death in adults with a severe form of *Plasmodium falciparum* (Peoc’h et al. 2000; Yapo Ette et al. 2002; Muehlethaler et al. 2005; Alunni-Perret et al. 2010; Prat et al. 2013; Yağmur et al. 2015). Many factors play specific but interlinked roles in the pathophysiology of CM, characterized by the sequestration of parasitized erythrocytes in the brain microvessels and the resulting metabolic and immune disorders. CM is considered to be a syndrome that includes the presence of asexual forms of *Plasmodium falciparum* in the blood smear, with no other etiology of encephalopathy (Idro et al. 2010). The criteria of malaria severity were first defined in 1990 by the World Health Organization (WHO), essentially concerning malaria in tropical areas, and then revised in 2000 (World Health Organisation (WHO) 2000), in 2010 (Word Health Organization (WHO) 2010), and again in 2015 (Word Health Organization (WHO) 2015). CM is considered a severe form of malaria infection with fatal outcomes.

Imported malaria is an infection that should be considered in the presence of any suggestive symptom (fever, chills, myalgias, asthenia, digestive problems, headaches, respiratory signs, and especially signs of severity) after returning from a stay in a malaria-endemic area (Bruneel et al. 2012). This was the case of the deceased who presented flu-like symptoms. Given the pandemic context and a negative PCR, he did not consider his symptoms to be important, although he came from an endemic malaria country (the transmission in Ivory Coast occurs throughout the year, with a peak incidence in April to July). It would appear that the deceased was not properly informed about prophylaxis nor was he aware of the warning signs of malaria. He did not seek pretravel health advice either. Neuromalaria occurs when infected red blood cells induce microvascular thrombosis in the cerebral vessels. It is fatal in almost 100% of cases in the absence of treatment, especially in nonimmune subjects (Alunni-Perret et al. 2010). Death may occur without prodrome, which gives this type of death a suspicious character requiring a forensic autopsy.

In our case, the external examination found mucocutaneous jaundice as was the case in an autopsy series of 18 sudden deaths due to severe malaria-discovered postmortem (Djodjo et al. 2015). Its incidence is reported to range from 11.5 to 62% (Djodjo et al. 2015). Its occurrence during severe malaria is either related to massive hemolysis or hepatocellular failure. Brain congestion and swelling were also reported in other case reports (Muehlethaler et al. 2005; Prat et al. 2013; Sevestre et al. 2021). This swelling is not associated with vasogenic edema, although cytotoxic edema is seen in some patients. The brain swelling is rather attributable to increased blood volume that occurs as a result of sequestration of the infected erythrocytes and/or an increase in cerebral blood flow, particularly in response to anemia, fever, and seizures (Mishra and Newton 2009). Hepatomegaly and splenomegaly are common in neuromalaria, due to the obstruction of the lobular veins of the liver and hyperplasia of the adenoid tissue (white pulp of the spleen) (Prat et al. 2013). Pulmonary edema, which causes adult respiratory distress syndrome during severe malaria, is most often associated with high plasmodial parasitemia (direct effect of sequestered parasites in the lungs) (Bhutani et al. 2020).

Histology is the main means of diagnosing malaria postmortem (Burel-Vandenbos et al. 2008; Alunni-Perret et al. 2010). The diagnosis is based on the observation of sequestrated parasitized red blood cells in the brain vessels. They may obstruct the lumen of small capillaries or be margined against the endothelium of larger vessels (Djodjo et al. 2015). These histological aspects reflect the properties of the parasitized red blood cells to adhere to the endothelial cells of capillaries and venules (Milner et al. 2015). Red blood cells acquire these cytoadherence properties when parasitized by mature forms of *Plasmodium falciparum* (Djodjo et al. 2015). The presence of malaria pigment is another important element of the histological diagnosis, as well as other histological findings (although inconsistent) such as ring hemorrhages around necrotic vessels and microthrombi (Milner et al. 2015). In our case, the diagnosis was based on the histological examination, by the demonstration of parasitized red blood cells obstructing the lumen of the cerebral capillaries and by the presence of malarial pigments in most of the organs sampled. Similar observations have been reported by other authors (Yapo Ette et al. 2002; Menezes et al. 2012).

Analysis of malaria case reports in the literature reveals the great scientific contribution of autopsies, as most of the data related to CM have been drawn from autopsy observations. Pathogenic mechanisms leading to cerebral malaria were at the beginning poorly defined as studies have been hampered by limited access to human tissues. This is also because limited studies can be performed in humans, and common models conducted on mouses do not reproduce all aspects of CM. The first attempts to discover the pathogenesis of this syndrome relied significantly on the histopathology of brain tissue from deceased CM patients (Rénia et al. 2012). According to Milner D., the diagnosis of CM can only be determined after death through postmortem examination of the brain and other organs (Milner 2020). Other malarial causes of death, such as severe malaria anemia, respiratory failure, acute respiratory distress syndrome, and acute renal failure, might be confirmed with laboratory testing without the need for an autopsy (Milner 2020). Indeed, macro- and microscopic examination of the human body after death allows the pathologist to catalog anatomic findings and determine an immediate cause of death, which allows malaria mortality to be averted (Milner 2020). This case also highlights the importance of malaria as the leading cause of unexplained death in the context of travel to endemic areas. There is no limit to the diagnosis of malaria in alive as well as dead people in endemic areas and among travelers visiting these areas.

## Conclusions

This article reports a case of CM-discovered postmortem. This case report illustrates the fact that travelers, often through the lack of advice, fail to take preventive treatment when making frequent trips to endemic malaria areas. It also confirms the importance of informing these travelers of the rapid fatal outcome and hence of the need for early treatment and follow-up. This report summarizes several features for the diagnosis of malaria and how postmortem investigations may lead to a retrospective diagnosis of a fatal complicated form, with cerebral involvement. The proper diagnosis was made possible, thanks to close collaboration between the different pathologists.

## Data Availability

Not applicable.

## Abbreviations

CM: Cerebral malaria
COVID-19: Coronavirus disease 2019
EDTA: Ethylenediamine tetraacetic acid
PCR: Polymerase chain reaction
RDT: Malaria rapid diagnostic test
WHO: World Health Organization
